# RNA-Seq Profiling of Intact and Enucleated Oocyte SCNT Embryos Reveals the Role of Pig Oocyte Nucleus in Somatic Reprogramming

**DOI:** 10.1371/journal.pone.0153093

**Published:** 2016-04-12

**Authors:** Lin Bai, Mengqi Li, Junli Sun, Xiaogan Yang, Yangqing Lu, Shengsheng Lu, Kehuan Lu

**Affiliations:** 1 State Key Laboratory for Conservation and Utilization of Subtropical Agro-bioresources, Guangxi University, No. 100 Daxue Road, Xixiangtang District, Nanning, Guangxi Zhuang Autonomous Region.530004, China; 2 Guangxi High Education Laboratory for Animal Reproduction and Biotechnology, Guangxi University, No. 100 Daxue Road, Xixiangtang District, Nanning, Guangxi Zhuang Autonomous Region.530004, China; Nanjing Agricultural University, CHINA

## Abstract

The specific molecular mechanisms involved in somatic reprogramming remain unidentified. Removal of the oocyte genome is one of the primary causes of developmental failure in cloned embryos, whereas intact oocyte shows stronger reprogramming capability than enucleated oocyte. To identify the reason for the low efficiency of cloning and elucidate the mechanisms involved in somatic reprogramming by the oocyte nucleus, we injected pig cumulus cells into 539 intact MII oocytes and 461 enucleated MII oocytes. Following activation, 260 polyploidy embryos developed to the blastocyst stage whereas only 93 traditionally cloned embryos (48.2% vs. 20.2%, *P* < 0.01) reached blastocyst stage. Blastocysts generated from intact oocytes also had more cells than those generated from enucleated oocytes (60.70 vs. 46.65, *P* < 0.01). To identify the genes that contribute to this phenomenon, two early embryos in 2-cell and 4-cell stages were collected for single-cell RNA sequencing. The two kinds of embryos were found to have dramatically different transcriptome profiles. Intact oocyte nuclear transfer embryos showed 1,738 transcripts that were up-regulated relative to enucleated cloned embryos at the 2-cell stage and 728 transcripts that were down-regulated (|log2Ratio| ≥ 5). They showed 2,941 transcripts that were up-regulated during the 4-cell stage and 1,682 that were down-regulated (|log2Ratio| ≥ 5). The most significantly enriched gene ontology categories were those involved in the regulation of binding, catalytic activity, and molecular transducer activity. Other genes that were notably up-regulated and expressed in intact oocyte nuclear transfer embryos were metabolic process. This study provides a comprehensive profile of the differences in gene expression between intact oocyte nuclear transfer embryos and traditional nuclear transfer embryos. This work thus paves the way for further research on the mechanisms underlying somatic reprogramming by oocytes.

## Introduction

Since 1997, the method of using somatic cell nuclear transfer (SCNT) to clone animals has received widespread recognition. Although SCNT has had great success in many species[1–8], there are still many issues to overcome, such as low efficiency, cloned embryo abortion, and newborn animal telomere loss [9]. In the process of SCNT, it is extremely critical that the cell be reprogrammed smoothly [10,11]. In a sense, the efficiency of cell reprogramming directly affects the efficiency of SCNT clone [12]. Epigenetic modifications of somatic nuclei transplanted into oocytes include imprinting wipes and activation of modifications which are coordinated with the development process. Some of these critical errors in reprogramming may be the reason why the rate of nuclear transplantation cloning is so low [13]. Pigs are domestic animals of profound and worldwide importance. The first cloned pig was born in 2000 by SCNT [14]. The technique of cloning pigs can be used as model organisms in regenerative medicine or in the closely related fields, such as genetic breeding and breed improvement.

In recent years, researchers have performed a great deal of research and achieved significant progress to address the low rate of nuclear transfer reprogramming [15]. Overexpressing a few key transcription factors can directly reprogram somatic cells into pluripotent stem cells, which are called induced pluripotent cells (IPS cells) [16]. After many experiments, results confirmed that IPS are inferior in quality to nuclear transfer embryonic stem cells (NT-ESCs), and only mouse IPSCs have been reported to fully develop into mature individuals [17,18]. An increasing number of studies have shown that some undefined maternal factors present in oocytes can reprogram somatic cells in a highly efficient manner, but these key factors have still not been identified[19–21]. With the continuous development and maturation of single-cell RNA sequencing (RNA-seq) technology, it is possible to elucidate the regulation of gene expression in cell development and reprogramming.

The ability of oocytes to undergo meiosis is determined by cytoplasmic factors, but after fertilization or activation of parthenogenesis, the ability of the former oocyte to undergo embryonic development largely depends on the oocyte nucleus. The nucleus does not play an important role in oocyte maturation, but it is very important for embryonic development and pronuclear formation [22]. In 2010, Jinsong Li et al. injected the cumulus cells into intact oocytes. After activation, 80% reconstructed embryos developed to the blastocyst stage, and ES cell lines were generated at a rate of 30% per reconstructed oocyte [15]. This confirmed that the mouse oocyte nucleus plays an important role in nuclear transfer embryonic development.

Pig is an important domestic animal and served as a model animal to assess the cellular reprogramming in large mammals. In the current study, we try to elucidate the influence of mechanical damage and other factors in the process of SCNT in embryonic development. Finally, single-cell RNA-seq was performed between early traditional SCNT embryos and intact oocyte SCNT embryos. Results showed the intact oocyte SCNT embryos to have advantages at the gene expression level. This study thus provides a foundation for further study of regulatory factors and signal pathways in the process of reprogramming.

## Materials and Methods

### Animal ethics

All animal procedures used in this study were carried out in accordance with the Guide for Care and Use of Laboratory Animals (8^th^ edition, released by the National Research Council, USA) and were approved by the Institutional Animal Care and Use Committee (IACUC) of Guangxi University. Pig ovaries for producing *in-vitro* matured oocytes and used as SCNT recipients were collected from ShiBu slaughterhouse in YongAn Village, Nanning, China. The breed of pig used to obtain embryos is Landrace-Yorkshire-Duroc.

### Production of enucleated and intact oocytes SCNT embryos

**Fig 1:**
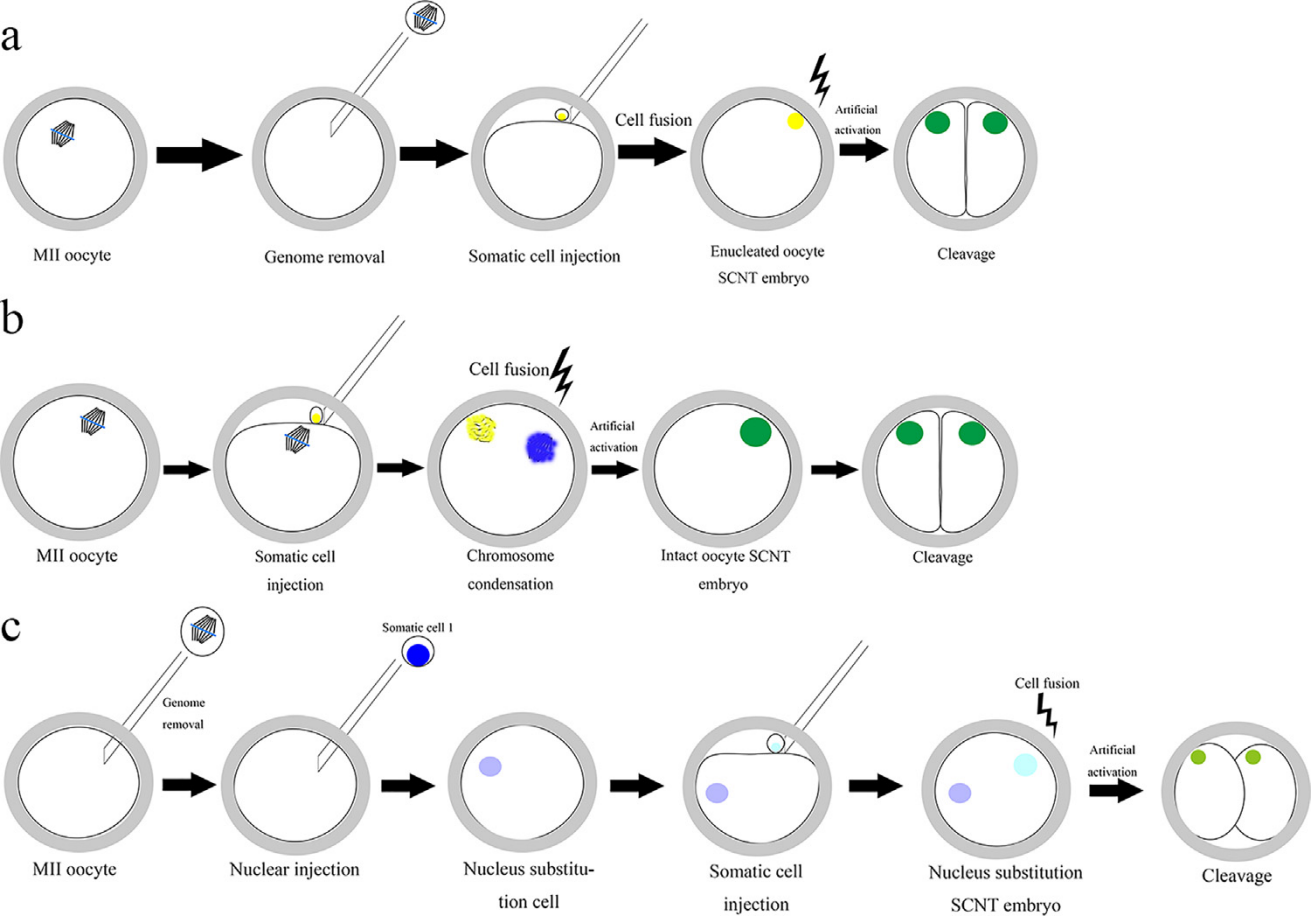
Schema of construction of traditional SCNT embryos(a), intact MII oocyte SCNT embryos(b) and chromosome exchange polyploid embryos(c).

SCNT with enucleated oocytes served as a traditional method in this study. Immature oocytes were collected from fresh pig ovary and subjected to in vitro maturation (IVM) to the metaphase II arrested stage. MII oocytes were enucleated using a sharp-crested pipette in a droplet of TL-Hepes-PVA medium containing 10% fetal bovine serum and 7.5 μg/ml CB. Cumulus cells served as donor cells. These were isolated from cumulus-oocyte complexes using hyaluronidase after IVM, and then injected into enucleated oocytes (S1 Fig). In SCNT with intact oocytes, cumulus cells were directly injected into MII oocytes (Fig 1). Two kinds of reconstructed oocytes were electrofused and activated using a single direct current pulse of 1200 V/cm for 30 μs. After electrofusion and activation, the reconstructed embryos were cultured in PZM-3 containing 7.5 μg/ml CB for 3 h to consolidate the effect. All organic and inorganic reagents were purchased from Sigma-Aldrich Co. (St. Louis, MO, USA)

### Chromosome preparation of blastocysts and cumulus cells

Blastocysts were incubated in 0.1 μg/ml demecolcine for 4–6 h, then incubated in 1% sodium citrate solution for 18–22 min at 37°C. Hypotonic solution-treated blastocysts were dropped onto clean slides and fixed in methanol: acetic acid (1:1 in volume) for 20 s, and methanol:acetic acid (3:1 in volume) for 10 min until dry, then stained with Giemsa for 10 min, and finally slides were air-dried.

Cumulus cells were cultured in 6 well cell culture cluster. After 2–3 days of culture, when the cells were in 70–80% confluence, they were incubated in 0.1 μg/ml demecolcine for 3–4 h. Subsequently, following trypsinization, the cells were resuspended in 0.075 M KCL solution for 20 min at 37°C. Fresh methanol:glacial acetic acid (1:3 in volume) was slowly added for prefixation. This fixation process was repeated three times. Finally, the cells were dropped onto -20°C precooled clean slides over one meter distance, after air drying, the cells were stained with Giemsa for 10 min.

### Assessment of blastocyst cell number

Day 6 blastocysts were washed three times in DPBS containing 0.1%BSA and fixed in 4% paraformaldehyde for 10 min. After fixation, blastocysts were again washed in BSA-DPBS. Subsequently the blastocysts were stained with 5 μg/ml Hochest 33342 in dark for 10 min and placed on clean slides within glycerin. This was followed by 3–5 rounds of washing in BSA-DPBS. Cell number in each blastocyst was counted under fluorescent microscope.

### Sample preparation for RNA-seq

In order to ensure that the genetic background of every sample was the same, the oocytes collected from a single ovary were cultured alone. All embryos were incubated in tyrode’s solution for 30–60 s to remove the zona pellucida at room temperature. Then the blastomeres were washed repeatedly in PBS containing 1% BSA to remove the tyrode and the cell debris. Finally, four blastomeres were collected in precooled cell lysis buffer in each group with a small volume of PBS. The total volume was kept within 5 μl and cells were stored at -80°C. The negative control was containing cell lysis buffer and BSA-PBS that was used to wash blastomeres.

### RNA amplification and sequencing library preparation

After cell lysis, DNase I was added to the total RNA to degrade contaminated DNA. Then oligo (dT) magnetic beads were used to enrich the mRNA, and mRNA was broken into short fragments with fragmentation buffer. Finally cDNA was synthesized using random hexamer-primer and mRNA fragments as templates. Sequencing adaptors were ligated to the fragments and the fragments were enriched by PCR amplification. Following quality inspection, the library products were ready for sequencing using Illumina HiSeq 2000. All of the RNA amplification and sequencing were finished in BGI company.

### Analysis of gene expression

The original image data produced by the sequencer were translated into raw reads, and these raw reads were preprocessed into clean reads by removing reads with adaptor sequences and low-quality reads for further analysis. Then clean reads were mapped to reference genomes using SOAP aligner[23]. Assessment of sequencing such as sequencing saturation, distribution of reads on reference genes was performed before further analysis.

The level of expression of each gene was determined using the numbers of reads uniquely mapped to the specific gene and the total number of uniquely mapped reads in the sample. Finally the genes that were differentially expressed among four samples were identified, and cluster analysis of gene expression patterns was performed using cluster software[24] and Java Treeview software[25].

With nr annotation, the Blast2GO program was used to get GO annotation of differentially expressed genes (DEGs). Then WEGO software [26]was used to classify these DEGs through their GO function and to meet the function of genetic variations from the macroscopic distribution characteristics. The calculating formula is as follows: $P=1-\sum_{i=0}^{m-1}\frac{\left(\begin{array}{c} M\\ i \end{array})(\begin{array}{c} N-M\\ n-i \end{array}\right)}{\left(\begin{array}{c} N\\ n \end{array}\right)}$ Here, N is the number of all genes with GO annotation; n is the number of DEGs in N; M is the number of all genes annotated to specific GO terms; m is the number of DEGs in M.

In vivo, genes usually play roles in biological functions by interacting with each other. For this reason, metabolic pathways and signal transduction pathways that were significantly enriched in DEGs were identified using the KEGG database[27] and compared to the overall genomic background.

### Real-time PCR

Real-time PCR reaction was done with 2×PCR MIX (Qiagen). Six random transcripts for validated were: DNMT1, FTL, POLR1D, RPS3, RPS20, and BUB3. The level of expression of each transcript was normalized to actin transcript levels. PCR was performed as follows: 95°C for 2 min to activate the Taq polymerase followed by 40 amplification cycles of 94°C for 10 s, 60°C for 10 s, and 72°C for 40 s. All reactions were performed in triplicate in a final volume of 16 μl containing 1 μl of cDNA used for RNA-seq and 0.2 μl of each primer. The relative expression levels were determined using the 2^-△△CT^ method.

## Results

### Pig oocyte nuclei can promote somatic cell reprogramming and SCNT embryo development

**Fig 2:**
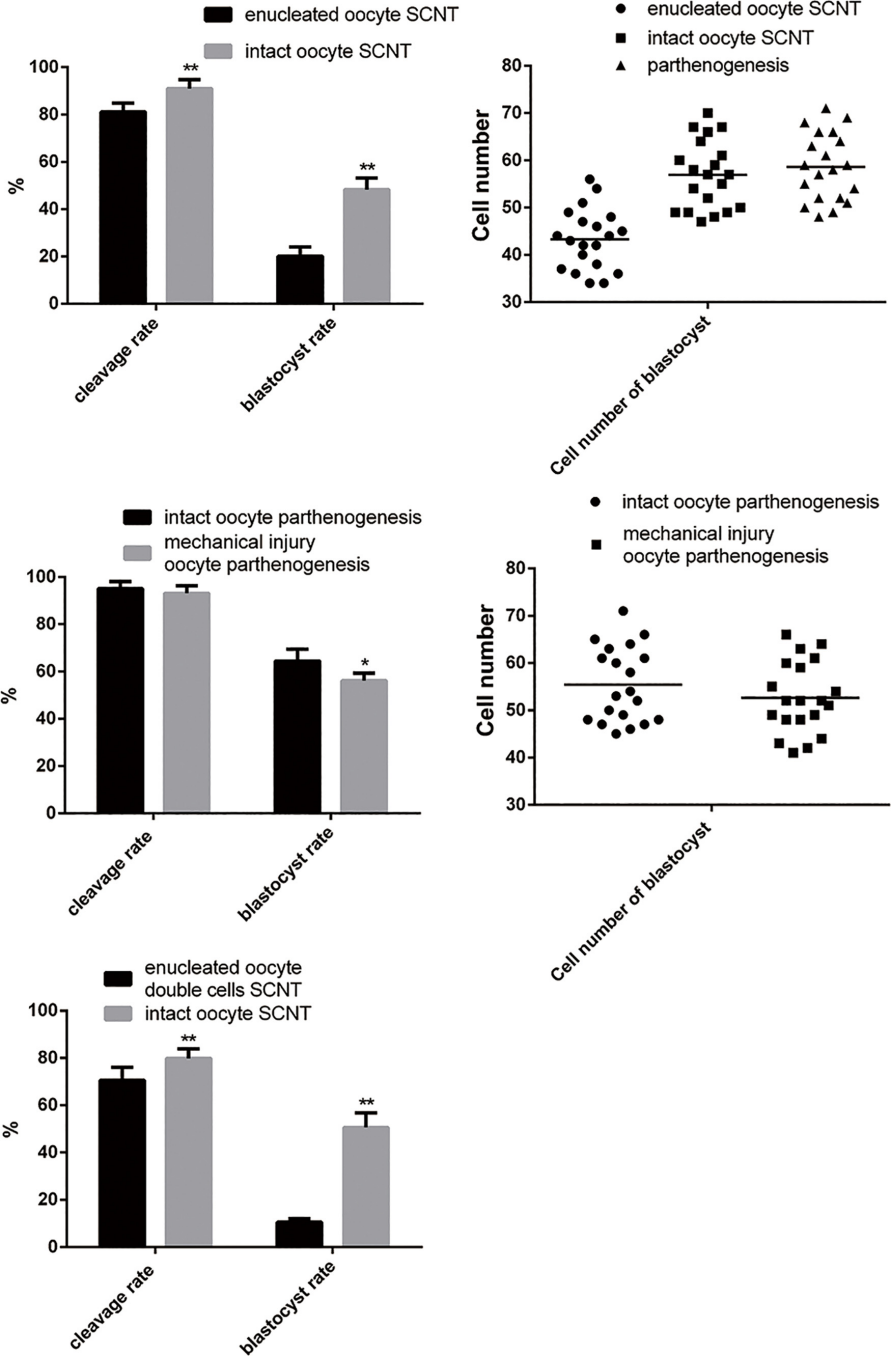
Graphical representations of the developmental capacity of traditional SCNT embryos and intact oocyte SCNT embryos. (a) Cleavage rate and blastocyst rate of enucleated oocyte SCNT and intact oocyte SCNT. (b) Number of cells in blastocysts generated from parthenogenesis, enucleated oocyte SCNT, and intact oocyte SCNT. (c) This cleavage rate and blastocyst rate in parthenogernesis are affected by the mechanical trauma associated with parthenogenesis. (d) The number of cells in blastocysts generated from intact oocyte parthenogenesis and mechanical trauma oocyte parthenogenesis. (e) The cleavage rate and blastocyst formation rate of intact oocyte SCNT after genome exchange.

In the experimental group, the somatic cells (cumulus cells) were directly injected into oocytes without removing the oocyte nucleus. After culturing in vitro, the multiploid embryo displayed developmental potentiality. These results showed the embryonic quality of intact oocyte SCNT to be better than that of enucleated oocyte SCNT. The cleavage rate increased from 81.15% to 91.05% and the blastocyst rate of the experimental group (48.29%) was significantly higher (by 28.14%) than that of control (20.15%) (Table 1)(Fig 2).

**Table 1:** Developmental potential of embryos reconstructed by injection of cumulus cells into intact and enucleated oocytes.

Type of experiments	No. of reconstructed oocytes	No. of cleavages(% of reconstructed oocytes)	No. of blastocysts(% of reconstructed oocytes)
Enucleated oocyte	461	374(81.15±3.705)^a^	93(20.15±3.797)^c^
Intact oocyte	539	475(91.05±3.78)^b^	260(48.29±4.932)^d^

Note: Significant differences showed with different superscripts in the same column (*P* < 0.05)

**Fig 3:**
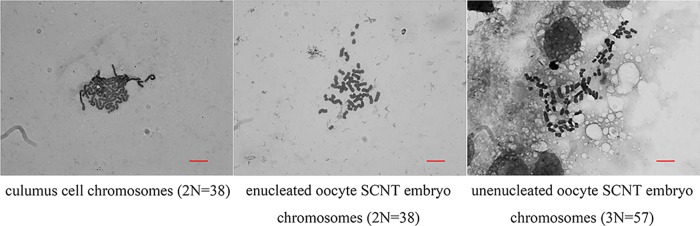
Karyotyping of cumulus cells, enucleated oocyte SCNT embryos and intact oocyte SCNT embryos.

The chromosome count of the multiploid embryos was 57, equal to the number in somatic cells (38) plus the number in oocytes (19), suggesting these embryos were triploid (Fig 3).

The quality of blastocysts produced here was assessed by determining the total number of cells in blastocysts following staining with Hochest33342. Parthenogenetic blastocysts served as the control group. Twenty blastocysts were counted in each group. The average number of cells in each parthenogenic blastocyst was found to be 58.60, and the average number in the enucleated oocyte SCNT group was found to be 43.30, markedly fewer than in control blastocysts. However, the blastocysts in the intact oocyte SCNT group had an average of 56.95 cells, only slightly fewer than in controls with significant differences(P<0.05)(Table 2).

**Table 2:** The cell number of blastocyst generated from parthenogenesis,enucleated oocyte NT and intact oocyte NT.

Type of experiments	No. of blastocysts	Cell number of blastocyst
**Parthenogenetic oocyte**	20	58.60±7.163^a^
**Enucleated oocyte NT**	20	43.30±6.392^b^
**Intact oocyte NT**	20	56.95±7.215^ac^

Note: Significant differences showed with different superscripts in the last column (*P* < 0.05)

**Fig 4:**
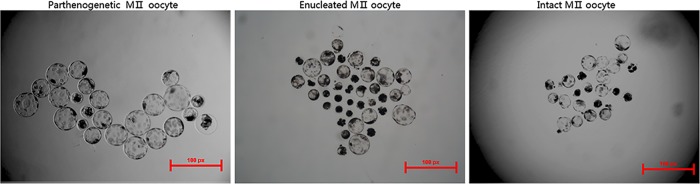
The shape of blastocysts generated from parthenogenesis, enucleated oocyte SCNT and intact oocyte SCNT.

Morphological analysis revealed the parthenogenetic blastocysts to be larger and more transparent than other blastocysts and to have a more uniform cell distribution (Fig 4). However, enucleated oocyte SCNT and intact oocyte SCNT blastocysts were both significantly different from parthenogenetic blastocysts. Their diameters were smaller, and they were darker in color. Most enucleated oocyte SCNT blastocysts differed from other blastocysts Moreover, a great number of intact oocyte SCNT blastocysts that were shaped like the numeral “8” showed better quality after hatching than other kinds of blastocysts.

### Mechanical injury to SCNT affect embryonic development

In the process of enucleation in SCNT, the glass pipette punctures the zona pellucida and enters the perivitelline space, while passing through the vitelline membrane it absorbs cytoplasm and membrane structure near the spindle. This process causes mechanical injury to the oocyte, even with a glass pipette of 10–15 μmin diameter. Nevertheless, during intact oocyte SCNT, there was no need to absorb the spindle, so there was no mechanical injury. For this reason, it was impossible to rule out the influence of the mechanical injury in the difference in development potential between conventional and intact oocyte embryos. For this purpose, this experimental simulation of spindle drain in the operation of SCNT was designed; however, the spindle structure was not removed, only injected back into the oocyte cytoplasm after aspiration to make sure there was no loss of nuclear matter, and the somatic cell nuclear injection step was skipped. The control was a simulated intact oocyte SCNT embryo, the somatic cell nuclear injection step was skipped, and the microinjection needle merely pricked a hole in the zona pellucida near the first polar body.

Then activation of parthenogenesis was performed on all those oocytes using the same parameters. Their cleavage and blastocyst rate efficiency were observed. After activation of parthenogenesis, the cleavage rate and blastocyst rate of mechanical injury group oocytes were lower than the group without mechanical injury group (cleavage rate: 93.136% vs. 95.032%, *P* ≈ 0.36; blastocyst rate: 56.14% vs. 64.42%, *P* ≈ 0.01), the average number of blastula cells was lower as well (52.65 vs. 55.40)(Table 3). However, the difference between the rates of blastocyst formation was only 8.28%. The blastocyst formation rates of enucleated and intact oocyte SCNT embryos (20.15% vs. 48.29%) were not comparable. This demonstrated that the process of removing the nucleus might cause some mechanical injury to the oocyte and have some but not significant negative effects on development of the embryo. Nearly half of intact oocyte cloned embryos developed to blastocysts. This is likely to be associated with other reasons not mechanical injury.

**Table 3:** The influence come from mechanical trauma to the cleavage rate and blastocyst rate and blastocyst cell number of parthenogenesis.

Type of experiments	No. of manipulated oocytes	No. of cleavages(% of manipulated oocytes)	No. of blastocysts(% of manipulated oocytes)	No. of blastocysts	Cell number of blastocyst
Intact oocyte parthenogenesis	418	397(95.032±3.022)	266(64.42±5.02)^a^	20	55.40±7.97
Mechanical trauma oocyte parthenogenesis	396	372(93.136±3.189)	227(56.14±3.18)^b^	20	52.65±7.53

Note: Significant differences showed with different superscripts in the same column (*P* < 0.05)

### Maternal nucleus in intact oocyte SCNT embryo cannot be replaced by somatic nucleus

There is approximately 2–10% polyspermy in human-assisted reproductive technology. The rates of mature oocytes and 2PN embryo formation were significantly improved after polyspermy. For this reason, the high blastocyst formation rate in the intact oocyte SCNT embryo might be associated with polyploidy. The issue of whether intact oocyte cloned embryos’ redundant chromosomes are the main cause of efficient somatic cell reprogramming or embryonic development was addressed here. Furthermore, to determine whether maternal chromosomes can be replaced by any other chromosomes, another polyploid embryo was constructed as follows: After removing the maternal nucleus, a somatic cell nucleus was injected into the oocyte to replace the maternal one, and then a second somatic cell was added to produce the polyploid embryo. Then the intact oocyte cloned embryos and reconstructed embryos were compared to determine whether the polyploid embryos had the same or similar viability.

Results showed that the blastocyst formation rate of reconstructed embryos after chromosome replacement only reached 10.5%, far below the blastocyst formation rate of intact oocyte cloned embryos (50.6%)(Table 4). It can be concluded that the increase in the number of chromosomes does not necessarily promote cell reprogramming or embryonic development and that the maternal nucleus in intact oocyte SCNT embryo cannot be replaced by somatic nucleus [28]. The source of the extra chromosomes (paternal/maternal sources) and the gene expression pattern in the embryo may play more important roles in embryo development than previously thought.

**Table 4:** The cleavage rate and blastocyst rate of intact oocyte NT after genome exchage.

Type of experiments	No. of reconstructed oocytes	No. of cleavages(% of reconstructed oocytes)	No. of blastocysts(% of reconstructed oocytes)
Intact oocyte NT	231	184(79.87±4.11)^a^	117(50.61±6.152)^c^
Enucleated oocyte double cells NT	209	148(70.57±5.56)^b^	22(10.50±1.542)^d^

Note: Significant differences showed with different superscripts in the same column (*P* < 0.05)

### Expression profiles of early porcine enucleated and intact oocyte SCNT embryos

For convenient description, the 2-cell stage embryo of the enucleated oocyte is referred to as A, and the 4-cell stage embryo of the enucleated oocyte is termed B. The 2-cell stage embryo of the intact oocyte SCNT has been referred to as C, and the 4-cell stage embryo of the intact oocyte SCNT is termed D. After the cDNA library was constructed, four samples’ library products were sequenced using Illumina HiSeqTM 2000.

Embryo A produced 36,857,696 total reads, embryo B 36,903,948 reads, embryo C 35,669,788 reads, and embryo D 36,381,536 reads. Among these total reads, there were 26,431, 727 total mapped reads for A, 27,759,843 for B, 26,776,345 for C, and 27,490,694 for D (Table 5). Among these four samples, the proportions of clean reads reached 98.5% of total reads, and the number of detected genes all tended to be saturated.

**Table 5:** Summary of mapping results of four samples (mapping to reference genome).

**Sample ID**	**Total Reads**	**Total Base Pairs**	**Total Mapped Reads**	**Perfect Match**
**A**	36,857,696 (100.00%)	1,806,027,104 (100.00%)	26,431,727 (71.71%)	20,801,269 (56.44%)
**B**	36,903,948 (100.00%)	1,808,293,452 (100.00%)	27,759,843 (75.22%)	16,678,992 (45.20%)
**C**	35,669,788 (100.00%)	1,747,819,612 (100.00%)	26,776,345 (75.07%)	20,895,257 (58.58%)
**D**	36,381,536 (100.00%)	1,782,695,264 (100.00%)	27,490,694 (75.56%)	20,636,355 (56.72%)
**Sample ID**	**< = 3 bp Mismatch**	**Unique Match**	**Multi-position Match**	**Total Unmapped Reads**
**A**	5,630,458 (15.28%)	23,010,280 (62.43%)	3,421,447 (9.28%)	10,425,969 (28.29%)
**B**	11,080,851 (30.03%)	24,853,359 (67.35%)	2,906,484 (7.88%)	9,144,105 (24.78%)
**C**	5,881,088 (16.49%)	23,222,457 (65.10%)	3,553,888 (9.96%)	8,893,443 (24.93%)
**D**	6,854,339 (18.84%)	23,214,726 (63.81%)	4,275,968 (11.75%)	8,890,842 (24.44%)

**Fig 5:**
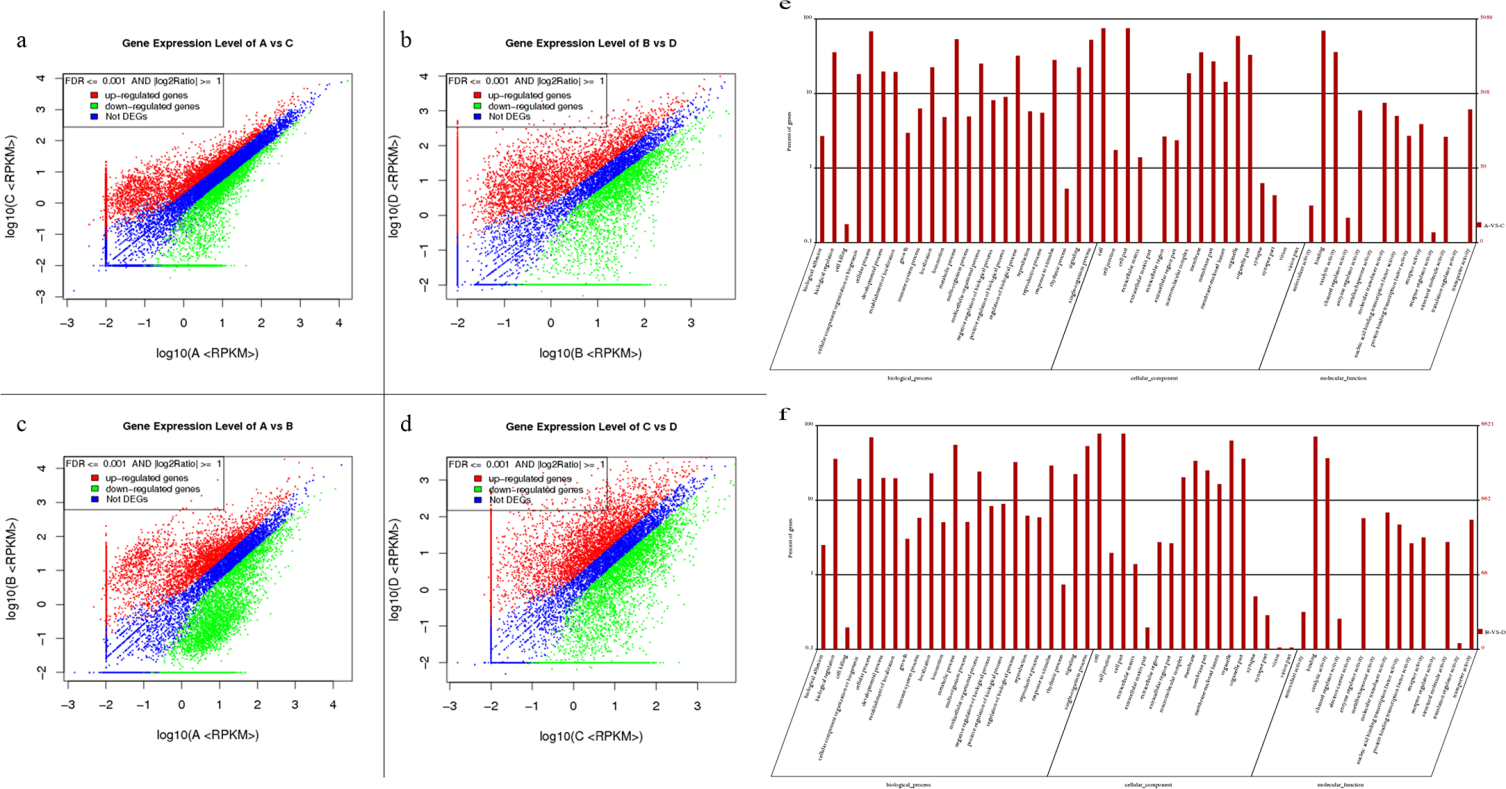
Scatter plot of DEGs in (a) A vs. C; (b) B vs. D; (c) A vs. B; (d) C vs. D and GO functional classification of DEGs in (e) A vs. C and (f) B vs. D. Note: For A vs. C, A is the control group. Red points indicate genes up-regulated in C relative to A, green points represent genes down-regulated in C relative to A, and blue points represent genes that showed no differences or fold change below 2.

This is the first work to show that the 2-cell stage of traditional enucleated oocyte SCNT embryo expresses roughly 28% of the total estimated genes in the porcine genome. Enucleated oocyte SCNT embryos at the 4-cell stage expressed roughly 22% of the total estimated genes. Intact oocyte SCNT embryos at the 2-cell stage expressed roughly 30% of the total estimated genes, and intact oocyte SCNT embryos at the 4-cell stage expressed roughly 27% of the total estimated genes (Fig 5).

### Differential gene expression between early porcine enucleated and intact oocyte SCNT embryos

**Fig 6:**
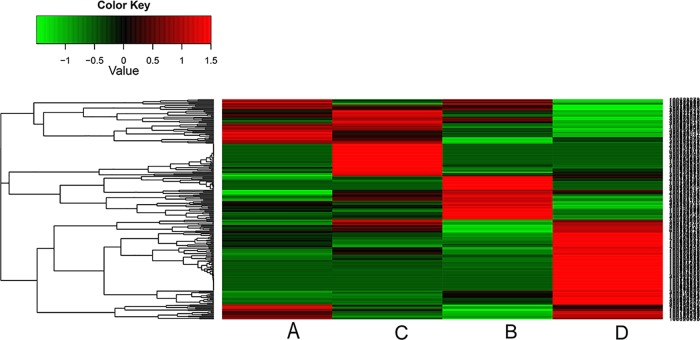
Cluster image of DEG levels of four samples. Each column represents an experimental sample, and each row represents a gene. Differences in expression are shown in different colors. Red indicates up-regulation and green represents down-regulation.

The embryos generated using two different techniques showed large differences in their level of transcription. Intact oocyte SCNT embryos at the 2-cell stage had 1,738 genes that were up-regulated relative to the enucleated SCNT embryo at the same stage and 728 that were down-regulated (|log2Ratio| ≥ 5). Intact oocyte SCNT embryos at the 4-cell stage had 2,941 genes that were up-regulated relative to enucleated SCNT embryos and 1,682 that were down-regulated (|log2Ratio| ≥ 5) (Fig 6). The genes expressed in two kinds of embryos revealed 566 unique genes expressed in enucleated oocyte SCNT embryos, and 1,256 unique genes expressed in intact oocyte SCNT embryos at the 2-cell stage, 1,163 unique genes expressed in enucleated oocyte SCNT embryo, and 1,699 unique genes expressed in intact oocyte SCNT embryo at the 4-cell stage. The top ten most expressed genes in the two embryos are shown in Table 6.

**Table 6:** Top 10 unique genes in gene expression profiles of four samples.

A		B		C		D	
Symbol	GO function	Symbol	GO function	Symbol	GO function	Symbol	GO function
LOC100156764	Cation binding	LOC100156727	Transmembrane signaling receptor activity	CRYBB1	Structural molecule activity	PDXK	Transition metal ion binding
LOC102162802	Nucleic acid binding	LOC102161209	---	FAM89B	---	TAC1	---
LOC102157643	Kinase binding	PLA2G4A	Phospholipid binding	ATP6V0E2	ATPase activity	LOC100512673	—
LOC102165660	Protein binding	LOC100628195	—	LOC100153329	Nucleic acid binding	OC100521510	Protein binding
LOC100515582	—	LOC100738735	Potassium channel activity	ATP5G1	Hydrogen ion transmembrane transporter activity	SCNM1	Cation binding
LOC102166678	Binding	DPY30	Identical protein binding	LOC102157944	—	PIGH	Acetylglucosaminyltransferase activity
LOC102166686	Binding	LOC100516621	Receptor binding	COX6B	Heme-copper terminal oxidase activity	FZD2	Protein-domain-specific binding
LOC100622308	Steroid binding	LOC102168186	—	TAF8	Nucleic acid binding	TIMM8A	Cation binding
LOC100737350	Enzyme binding	IPO8	Ras GTPase binding	LOC102158891	—	MRPL42	Structural molecule activity
PLDN	Syntaxin binding	LOC100624598	—	GSTP1	Transferase activity	IL2RG	Growth factor binding

However, the top ten genes of B and D, showed no genes whose molecular function was nucleotide binding, unlike those of A and C. Hence, it is plausible that, in nuclear transfer embryos, 2-cell and 4-cell embryos have considerably different transcription activity. DNA transcription was more common in 2-cell embryos, and material transport across the membrane was more common in 4-cell embryos.

WEGO analysis of differentially expressed genes between two kinds of embryos at the 2-cell stage showed that most of the genes to be involved in metabolic processes related to various cellular organic substances, and their central molecular functions were heterocyclic compound binding and organic cyclic compound binding and so on. The situation at the 4-cell stage was the same as the 2-cell stage. The most pronounced degree of enrichment of any gene cluster were binding regulation, catalytic activity, and molecular transduction activity, respectively. Other genes appeared up-regulated in intact oocyte SCNT embryos were mostly involved in various metabolic processes.

### Pathways in category-specific modules between early porcine enucleated and intact oocyte SCNT embryo

**Fig 7:**
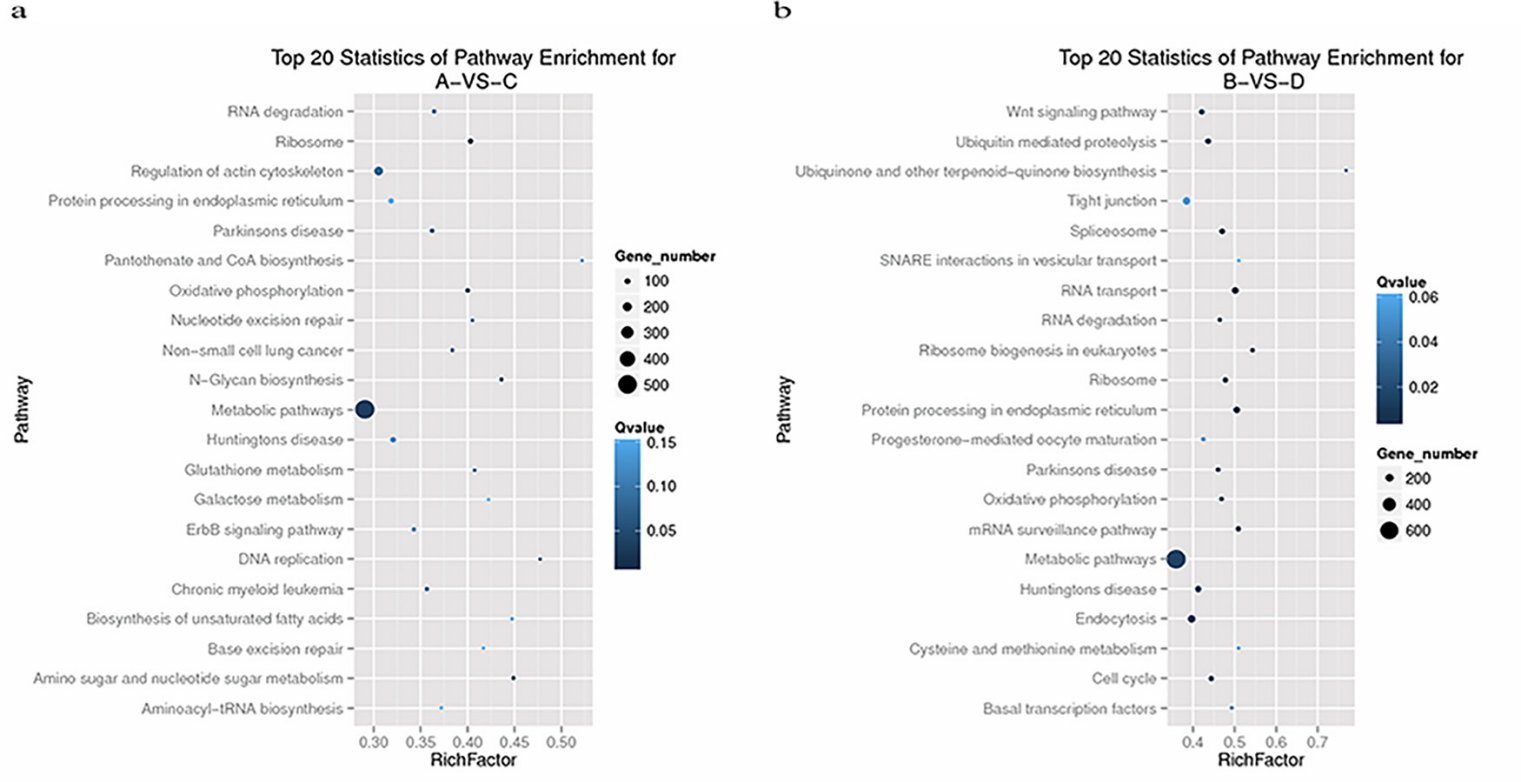
Top 20 statistics of pathway enrichment for (a) A vs. C and (b) B vs. D.

Genes usually interact with each other to play roles in certain biological functions. Pathway-based analysis can help further elucidateing the biological functions of genes. We found out ten most meaningful pathways through the enrichment analysis of differentially expressed genes between two kinds of embryos in 2-cell stage (Fig 7A). They were as follows: 1. Ribosomes, 2. oxidative phosphorylation, 3. amino sugar and nucleotide sugar metabolism, 4. N-glycan biosynthesis, 5. metabolic pathways, 6. DNA replication, 7. Parkinson's disease, 8. glutathione metabolism, 9. nucleotide excision repair, 10. RNA degradation.

Ribosomes are an important site of protein synthesis in cells. Proteins with signal sequences are synthesized in ribosomes that are attached to the endoplasmic reticulum. Ribosomes have 60s and 40s ribosomal subunits. These are constructed in the nucleolus and then exported.

Oxidative phosphorylation is the primary form of ATP synthesis in the body, and it provides a usable energy source for the life activities of aerobic cells. It consists of the release of energy from eukaryotic cells and plays an important role in activities that require large amounts of energy, such as cell proliferation and differentiation. In many examinations of RNA-sequencing in eukaryotic cells, oxidative phosphorylation was also found to be the most common gene enrichment pathway (S2 and S3 Figs).

At the same time, the most meaningful pathways of DGE in 4-cell stage were as follows (Fig 7B): 1.RNA transport, 2. protein processing in endoplasmic reticulum, 3. ribosome biogenesis in eukaryotes, 4. mRNA surveillance pathway, 5. spliceosome, 6. ribosome, 7. ubiquitin mediated proteolysis, 8. oxidative phosphorylation, 9. Parkinson's disease, 10. cell cycle.

The ribosome and oxidative phosphorylation pathways were down-regulated in enucleated oocyte SCNT embryos and up-regulated in intact oocyte SCNT embryos, and many more genes involved in these processes appeared significantly up-regulated (S4 and S5 Figs).

The signal path of protein processing in endoplasmic reticulum showed visible enrichment in the differentially expressed genes of the KEGG pathway in 4-cell embryo. The endoplasmic reticulum is near the nucleus and has many ribosomes on its surface. During nuclear transfer, the endoplasmic reticulum may also be removed. The endoplasmic reticulum is an important part of the cytoplasm membrane system. It connects cell membrane to the outer membrane of the nucleus and keeps the nucleus, cytoplasm, and cell membrane connected to each other. All of the cell's normal protein translation starts in the endoplasmic reticulum, and many materials from the nucleus that are transferred to the cytoplasm, cell membrane, and extracellular matrix require the help of the endoplasmic reticulum.

For this reason, it is here speculated that the deficiency of protein processing in the endoplasmic reticulum, other process in the ribosome, and oxidative phosphorylation may be the primary causes of the developmental disadvantages that enucleated oocyte SCNT embryo showed relative to intact oocyte SCNT embryo (S6 Fig).

Through the comparison of gene expression in two kinds of SCNT embryos at the same developmental stage, it was here preliminarily concluded that there are huge differences in gene expression in the enucleated and intact oocyte SCNT embryos. During embryonic development, the number of differentially expressed genes gradually increased. In early intact oocyte SCNT embryo, processes related to cellular organic substances showed more activity, material transport across the membrane was more frequent, and the nucleic acid transcription began to weaken after the 2-cell stage.

### Expression pattern of transcription activity genes in the nucleus

**Fig 8:**
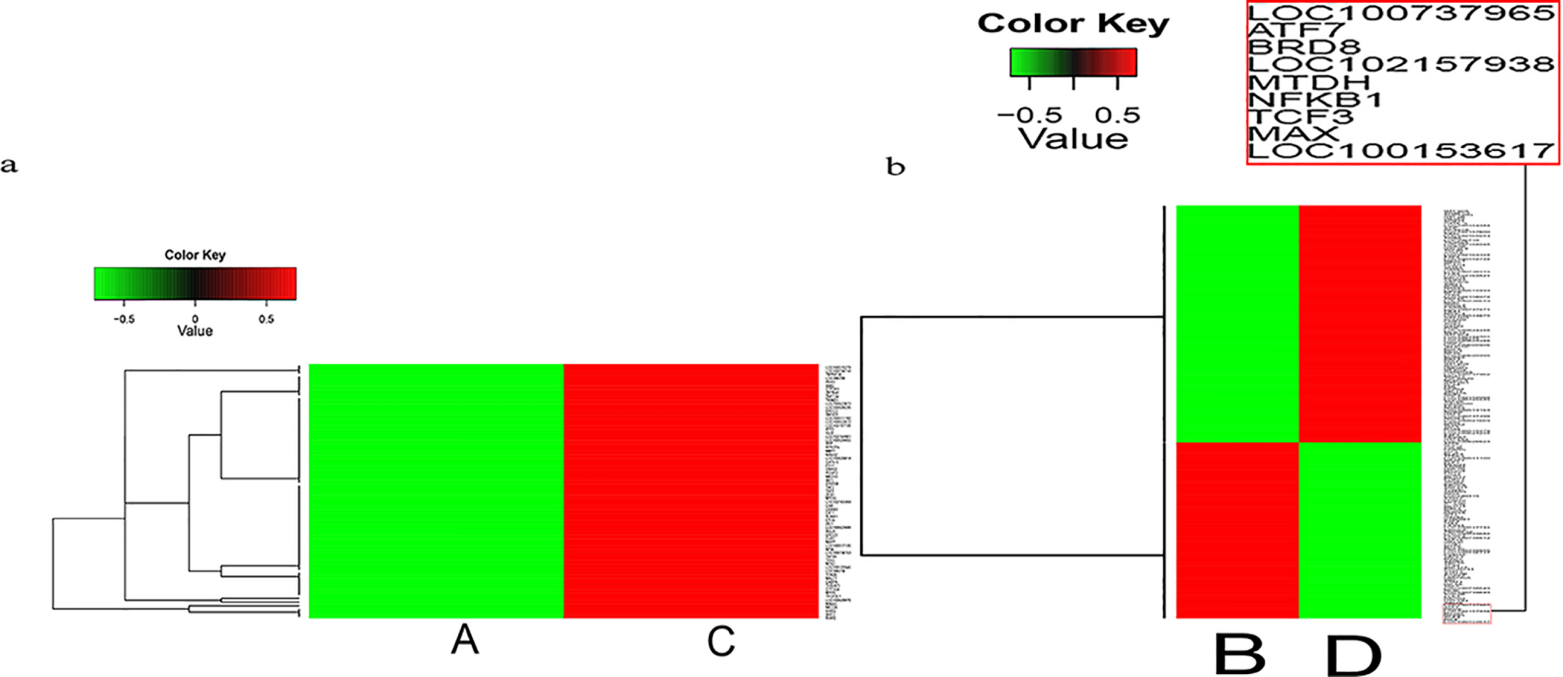
Cluster image of DEG levels of transcription factor activity genes expressed in the nucleus. Each column represents one sample, and each row represents one gene. Red indicates up-regulation and green indicates down-regulation.

Genes with similar expression patterns usually had some functional correlation. Generally, cell reprogramming is associated with transcription factors. For this reason, 267 genes with transcription activity expressed in nucleus were screened from global DGEs between two reconstructed NT embryos. Here, 70 transcription activity genes were differentially expressed in two embryos at the 2-cell stage, and 197 transcription activity genes were differentially expressed at the 4-cell stage. Most of these genes showed faint expression in enucleated oocyte NT embryos but had a significant up-regulation in intact oocyte NT embryos during the same stage. Cluster analysis of these gene expression patterns was performed using cluster software[24] and Java Treeview software[25] (Fig 8).

Almost all genes with transcription activity showed significant up regulation in intact oocyte SCNT embryos at the 2-cell stage. A small number of genes with transcription activity showed down-regulation in intact oocyte SCNT embryos at the 4-cell stage, but some of these genes acted as negative regulators during cell development. These included MAX, TCF3, and NFKB1. Scientists speculated that Max is a repressor of germ cell-specific genes in mouse ES cells[29]. It interacts with histone H3K9 methyltransferases, knockdown of Max results in a decrease in histone H3K9 dimethylation at their promoter regions and causes selective, global derepression of germ-cell-specific genes [29]. Tcf3 is a repressor of Nanog gene. It is necessary to limit the steady-state levels of Nanog promoter’s activity in self-renewing ES cells [30]. It binds to a promoter regulatory region of Nanog gene and represses its transcriptional activity through a Groucho interaction domain-dependent process. NF-κB has a function in the regulation of inflammation, oncogenesis, and human cancer [31,32]. It regulates the expression of genes involved in development and progression of cancer such as proliferation, migration, and apoptosis. Aberrant NF-kB activation has been detected in many human malignancies. Disruption of Nfkb1 inhibits skeletal muscle atrophy[33].

### Real-time RT-PCR validation of RNA-seq results

**Fig 9:**
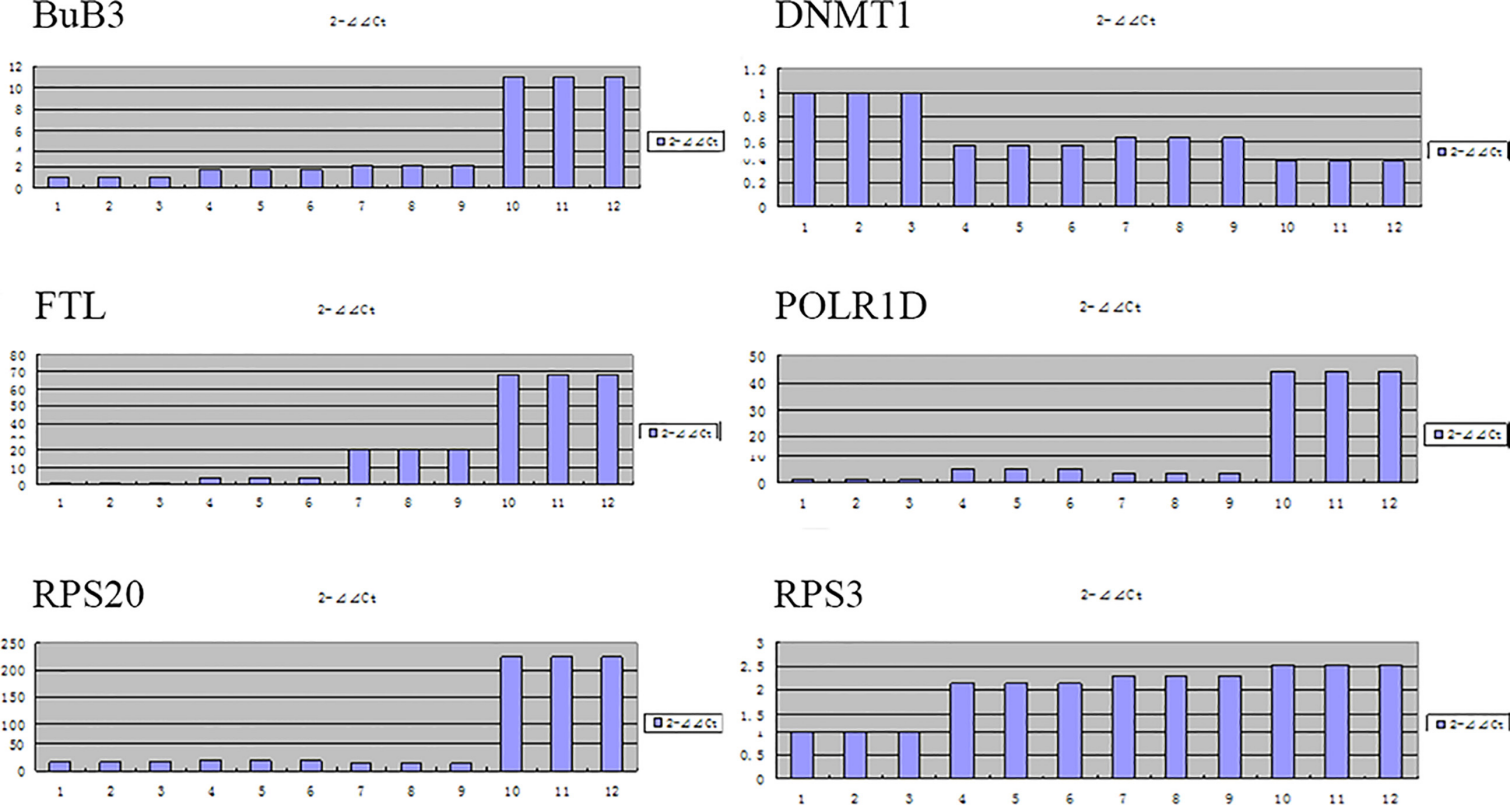
Expression of selected genes in real-time quantitative RT-PCR.

Six genes with high expression quantity were randomly selected: DNMT1, FTL, POLR1D, RPS3, RPS20, and BUB3 for QRT-PCR detection (Tables 7 and 8). Then we made a comparison between test results and RNA-seq results, and found that the expression trend of these six genes were basically identical under two kinds of detection methods (Fig 9).

**Table 7:** Primer sequences used in real-time RT-PCR.

Gene	Sense primer 5′–3′	Antisense primer 5′–3′
BUB3	CCCAACACCTCCCAGTTCCT	TGCGTTGGATCGTAGAAGGC
POLR1D	CCAGCTGTTGAGCCCTTTCA	AGGTGGACTCCTTTCTGCCG
DNMT1	AAATTTGGTGGTAGCGGACG	GGTGACGGCATCTCTGGAAT
FTL	TCCAGGACGTGCAGAAACCA	AGCGCATGCAGATCCACAAG
RPS20	GGATTCGGATCACCCTCACC	CACCGGTCCCTTCACTTTGA
RPS3	TGCGGTTCATCATGGAGAGT	GTCCCCACTGTGGATCATCA

**Table 8:** Expression of selected genes in RNA-seq.

Symbol	GeneID	A-(RPKM)	B-(RPKM)	C-(RPKM)	D-(RPKM)
BUB3	100,156,053	100.3058	129.4132	140.4	336.4103
DNMT1	606,746	4536.711	1164.929	4510.403	162.8543
FTL	397,035	2821.996	10,302.17	16,819.9	62,659.76
POLR1D	100,627,477	585.2484	1503.338	801.5405	5156.534
RPS20	414,395	653.8939	4867.138	3316.784	13,925.71
RPS3	733,671	292.8508	322.1036	331.8985	605.6551

## Discussion

Traditional nuclear transfer embryos usually suffer developmental blockage caused by abnormal transcription even if the recipient is a zygote [34]. Upon the use of better-quality oocytes for SCNT, the developmental potential of the reconstructed embryos were found to be improved and the blastocyst formation rate increased, but blockage of cleavage was still frequent[35–37]. When oocyte and somatic genomes exist in the same embryo, both of them stay in same environment, and the question of which of them will need to be expressed preferentially and whose pluripotent genes will be transcribed determines the epigenetic memory of the embryo[34].

Here, four embryos that had been constructed from enucleated oocyte SCNT and intact oocyte SCNT during the maternal-zygotic transition period and first cleavage period were subjected to RNA-sequencing, and the bioinformatics analysis. A significant difference was observed between the two kinds of embryos both during the first cleavage period and MZT period. Here, 267 genes related to transcriptional activity related genes in differentially expressed genes (DEGs) were found to be expressed in the nucleus, and most of them showed faint expression in enucleated oocyte SCNT embryos but were significantly up-regulated in intact oocyte SCNT embryos.

WEGO analysis of DEGs showed that the deepest enrich gene clusters were focus on binding regulation, catalytic activity, and molecular transduction activity. The other genes that appeared to be up-regulated in intact SCNT embryos were involved in various metabolic processes, organic metabolic activity, transmembrane transport, and amino sugar and nucleotide sugar metabolism. This appeared more intense during the early stage of intact oocyte SCNT embryos. DNA transcription abated after the 2-cell stage, and the most up-regulated genes were involved in heterocyclic compound binding and organic cyclic compound binding.

The ribosome and oxidative phosphorylation pathways showed up-regulation in intact oocyte SCNT embryos but not in enucleated oocyte SCNT embryos. Microarrays of mouse embryos during the 1–2-cell stage, showed strong signals related to the oxidative phosphorylation pathway[38]. This indicates that the oxidative phosphorylation and ribosome signal pathways play a key role in somatic reprogramming in mice and early nuclear transfer embryonic development. Research that regards the manner in oxidative phosphorylation regulating the cell cycle is under way.

It is here speculated that the deficiency of protein processing in the ribosomes and oxidative phosphorylation may be the primary reasons why enucleated oocyte SCNT embryos had such disadvantages in development relative to intact oocyte SCNT embryos.

Three signal pathways, those associated with ribosomes, oxidative phosphorylation, and the processing of proteins associated with the endoplasmic reticulum allowed precise control of the cell cycle and somatic cell reprogramming in SCNT embryonic development needs to be studied further.

Through the manner of reprogramming large mammal’s somatic cells, it is possible to generate multiploid ES cells. It may be possible to make genetically tailored tissues for transplantation in the near future and provide valuable materials for regenerative medicinal treatment, for research into somatic cell reprogramming associated with regenerative medicine, and for the treatment of degenerative diseases and organ transplantation research.

## Supporting Information

S1 Fig Schematic of somatic cell transfer without removal of the oocyte nucleus (PB1: first polar body; CC: cumulus cell). (TIF)

S2 Fig Genes in ribosome pathway differentially expressed between enucleated and intact oocyte NT 2-cell embryos. (TIF)

S3 Fig Genes in ribosome pathway differentially expressed between enucleated and intact oocyte NT 4-cell embryos. (TIF)

S4 Fig Genes in oxidative phosphorylation pathway differentially expressed between enucleated and intact oocyte NT 2-cell embryos. (TIF)

S5 Fig Genes in oxidative phosphorylation pathway differentially expressed between enucleated and intact oocyte NT 4-cell embryos. (TIF)

S6 Fig Genes in protein processing in endoplasmic reticulum pathway differentially expressed between enucleated and intact oocyte NT 4-cell embryos. (TIF)

## Abbreviations

SCNT: Somatic cell nuclear transferuclear
PA: Parthenogenetic activation
MZT: Maternal-zygotic transition
EGA: Embryonic gene activation
RNA-seq: RNA sequencing
IVM: In vitro maturation
DEG: Differente xpression genes
IVF: In vitro fertilization
