# Supplementary figures and images for: RNA-Seq Profiling of Intact and Enucleated Oocyte SCNT Embryos Reveals the Role of Pig Oocyte Nucleus in Somatic Reprogramming
Source: PLoS One. 2016 Apr 12;11(4):e0153093. doi: 10.1371/journal.pone.0153093 (PMC4829232; doi:10.1371/journal.pone.0153093)

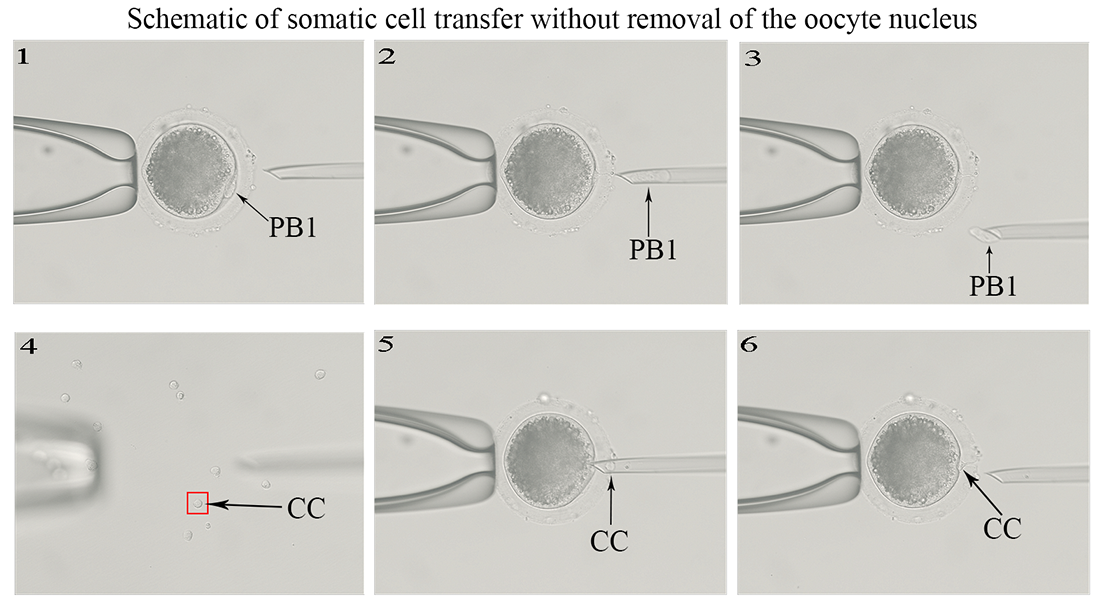

Supplement: S1 Fig — (TIF) [file pone.0153093.s001.tif]

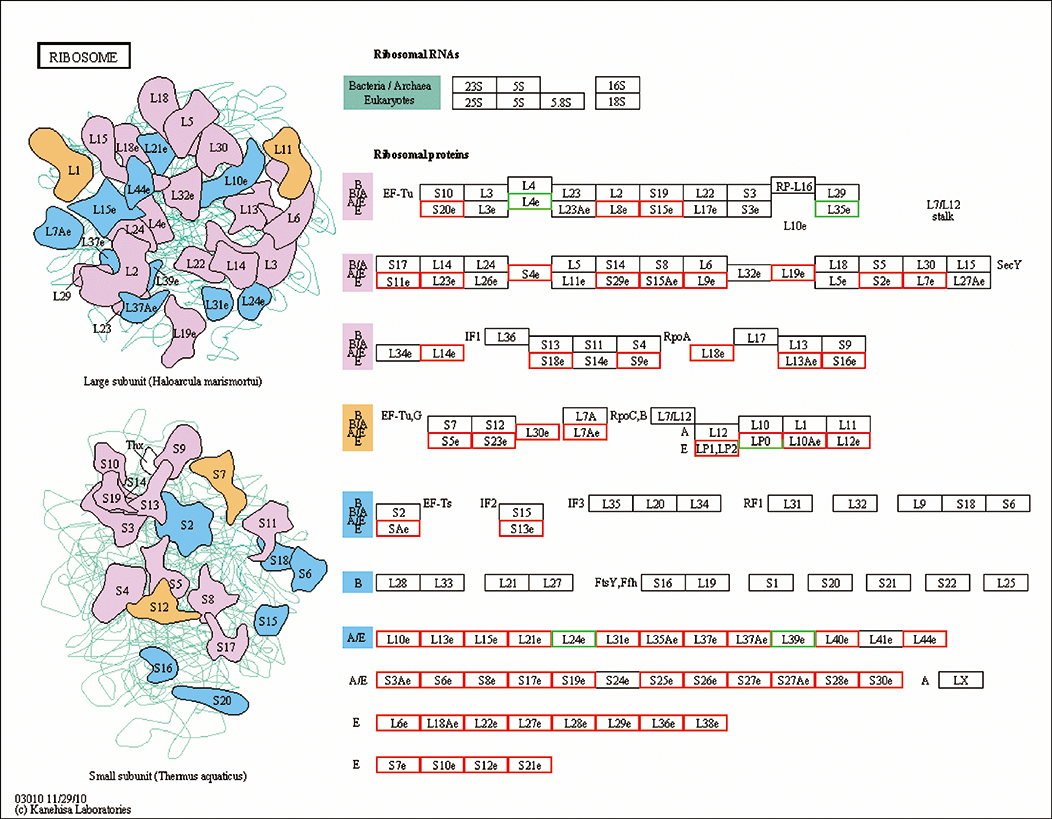

Supplement: S2 Fig — (TIF) [file pone.0153093.s002.tif]

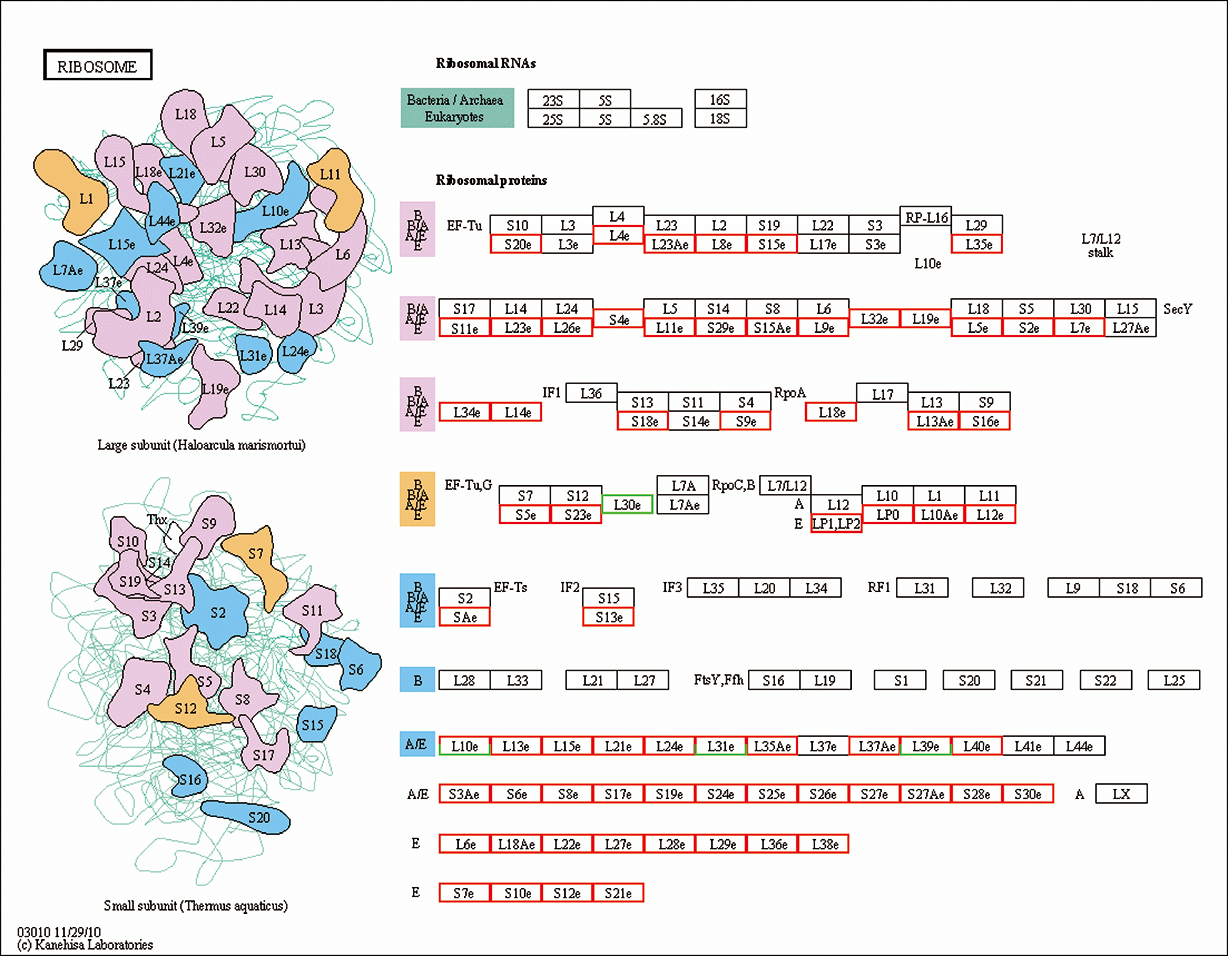

Supplement: S3 Fig — (TIF) [file pone.0153093.s003.tif]

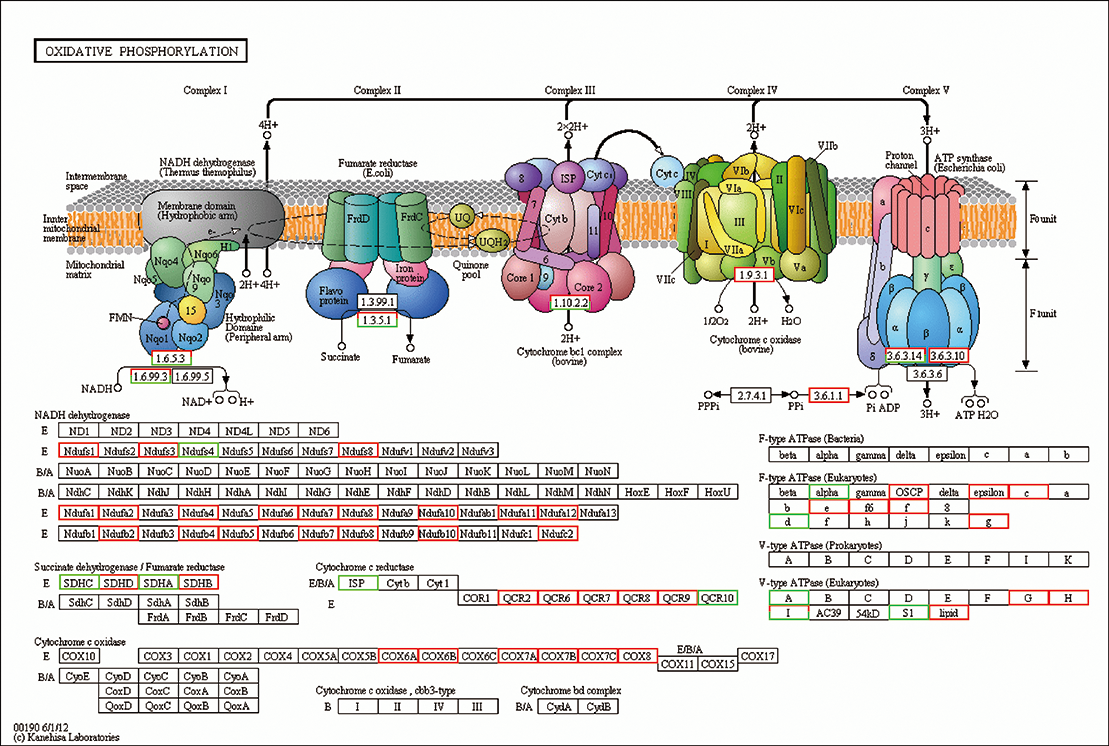

Supplement: S4 Fig — (TIF) [file pone.0153093.s004.tif]

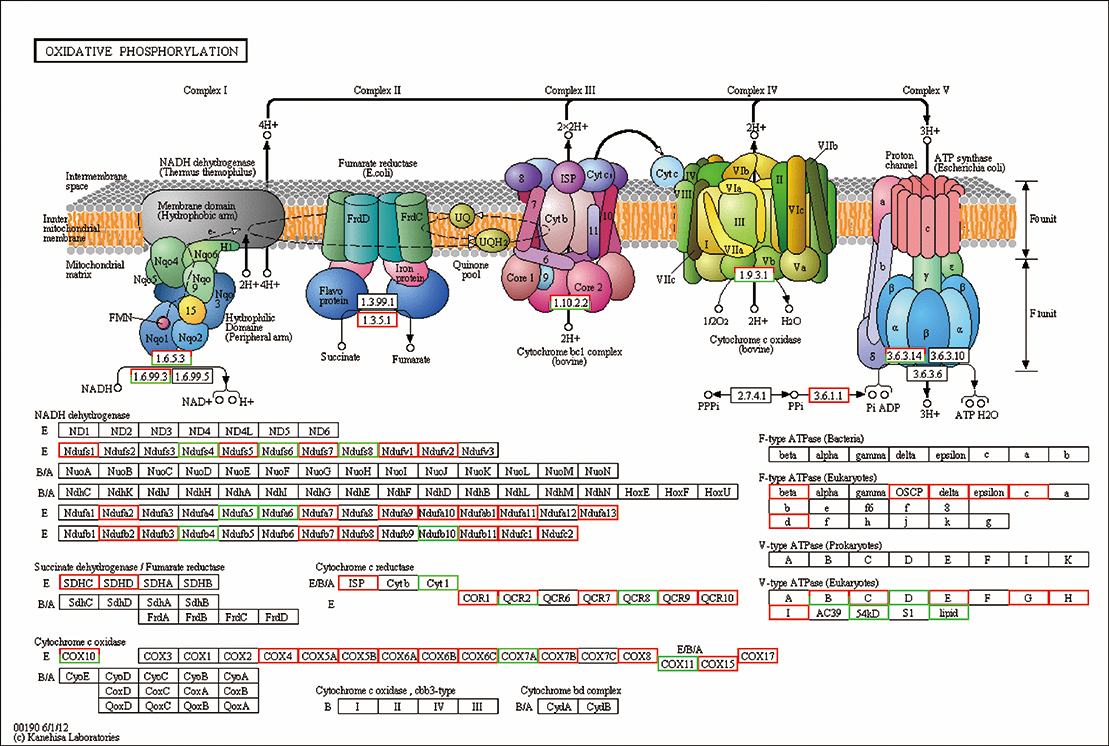

Supplement: S5 Fig — (TIF) [file pone.0153093.s005.tif]

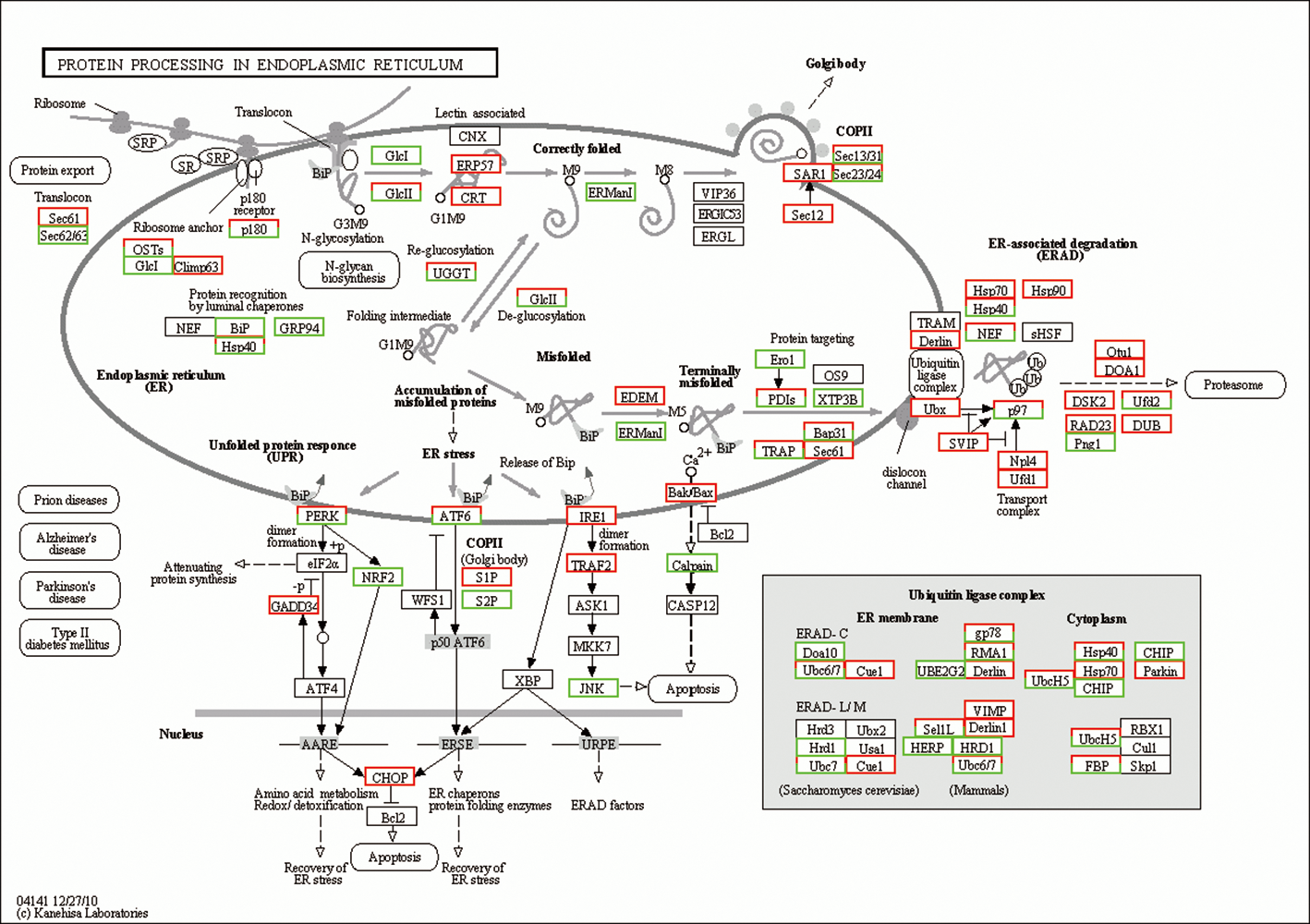

Supplement: S6 Fig — (TIF) [file pone.0153093.s006.tif]
